# Effect of miR-146a/bFGF/PEG-PEI Nanoparticles on Inflammation Response and Tissue Regeneration of Human Dental Pulp Cells

**DOI:** 10.1155/2016/3892685

**Published:** 2016-01-24

**Authors:** Lu Liu, Shan Shu, Gary Shunpan Cheung, Xi Wei

**Affiliations:** ^1^Operative Dentistry and Endodontics, Guanghua School of Stomatology, Affiliated Stomatological Hospital, Guangdong Province Key Laboratory of Stomatology, Sun Yat-sen University, Guangzhou, Guangdong 510055, China; ^2^Conservative Dentistry, School of Dentistry, The University of Hong Kong, Pok Fu Lam, Hong Kong

## Abstract

*Introduction*. Inflammation in dental pulp cells (DPCs) initiated by Lipopolysaccharide (LPS) results in dental pulp necrosis. So far, whether there is a common system regulating inflammation response and tissue regeneration remains unknown. miR-146a is closely related to inflammation. Basic fibroblast growth factor (bFGF) is an important regulator for differentiation.* Methods*. To explore the effect of miR-146a/bFGF on inflammation and tissue regeneration, polyethylene glycol-polyethyleneimine (PEG-PEI) was synthesized, and physical characteristics were analyzed by dynamic light scattering and gel retardation analysis. Cell absorption, transfection efficiency, and cytotoxicity were assessed. Alginate gel was combined with miR-146a/PEG-PEI nanoparticles and bFGF. Drug release ratio was measured by ultraviolet spectrophotography. Proliferation and odontogenic differentiation of DPCs with 1 *μ*g/mL LPS treatment were determined.* Results*. PEG-PEI prepared at N/P 2 showed complete gel retardation and smallest particle size and zeta potential. Transfection efficiency of PEG-PEI was higher than lipo2000. Cell viability decreased as N/P ratio increased. Drug release rate amounted to 70% at the first 12 h and then maintained slow release afterwards. Proliferation and differentiation decreased in DPCs with LPS treatment, whereas they increased in miR-146a/bFGF gel group.* Conclusions*. PEG-PEI is a promising vector for gene therapy. miR-146a and bFGF play critical roles in inflammation response and tissue regeneration of DPCs.

## 1. Introduction

Dental pulp cells (DPCs) are clonal precursors of dental pulp tissue, possessing self-renewal and multilineage differentiation capability. DPCs could differentiate into odontoblasts, adipocytes, chondrocytes and neurons, and so forth [1]. Lipopolysaccharide (LPS), one of the central regulators of pulpal pathogenesis, can bind to Toll-like receptor 4 (TLR4), to initiate inflammatory cascade through mediating synthesis and secretion of various inflammatory cytokines, causing damage or even necrosis of dental pulp [2]. Vital pulp provides biological defense and maintains pulp pressure against infection. However, once the inflammation spreads in pulp chamber and irreversible pulpitis establishes, the success rate of vital pulp therapy (VPT) decreases and saving teeth becomes very difficult [3]. Thus we speculate whether there is a mechanism to repress inflammation response and enhance tissue regeneration.

miR-146a is a newly discovered small RNA molecule closely related to inflammation and immune diseases. miR-146a is predicted to base-pair with sequences in the 3′UTR of the IL-1 receptor associated kinase 1 (IRAK1) and TNF receptor associated factor 6 (TRAF6) and acts as a fine tuning adaptor to prevent overstimulation of inflammatory response [4, 5]. Basic fibroblast growth factor (bFGF) is involved in multiple biological processes including cell survival, tissue regeneration, wound healing, inflammation, and immune response [6]. bFGF is an important factor for stimulating proliferation and differentiation of DPCs, participating in reparative dentin formation and repair of dental pulp [7]. Nevertheless, bFGF with a short half-life value is unstable and easy to degrade* in vivo*. Thus maintaining appropriate release concentration and time of bFGF through a delivery system is necessary [8].

Successful gene delivery relies on effective gene delivery vector. Viral vectors with high transfection efficiency are commonly applied for gene delivery. Nevertheless, viral vectors have side effects including immunogenicity, complicated synthesis procedure, and carcinogenicity [9]. In recent years, nonviral gene vectors with low immunogenicity, simplicity of synthesis, and ease of large-scale production have overcome problems occurring with viral vectors and attracted more attention [10]. PEI, a common polycation, possesses low cytotoxicity, high transfection efficiency, and pH buffering capacity. PEI is commonly applied for drug delivery, coating magnetic nanoparticles or transfection agent [11, 12]. Polyethylene glycol-polyethyleneimine (PEG-PEI) possesses superior property of reduced cytotoxicity, increased transfection efficiency, improved cell viability and polymer solubility, prolonged circulation time, and decreased nonspecific interaction with serum proteins [11]. Alginate, a linear heteropolysaccharide with high biocompatibility, is often used to generate hydrogel for the delivery of drugs and proteins [13]. The fine fabricated three-dimensional PEG-PEI alginate hydrogel could mimic natural microenvironment, deliver miRNAs and proteins to the cells, and promote cell-cell and cell-matrix interaction [14].

In our previous study, we revealed that Oct4B1 played a critical role in inflammatory response of DPCs through interaction with miRNAs [15]. However, so far little is known about the mechanism underlying the effect of miR-146a/bFGF on inflammation response and tissue regeneration of DPCs, as well as the potential application of PEG-PEI alginate hydrogel in dental regeneration. Therefore, in this study, we investigate the modulatory role of miR-146a and bFGF in DPCs with inflammation response. Alginate gel was used as a carrier to combine miR-146a/PEG-PEI nanoparticles and bFGF and explore their effect on cell proliferation and odontogenic differentiation of DPCs treated with LPS. The data generated from the present study will open novel possibility to repress inflammatory reactions and enhance regeneration, which would make it possible to replace current endodontic strategies with tissue engineered dental pulp.

## 2. Materials and Methods

### 2.1. Isolation and Expansion of Human DPCs

Normal human premolars and impact third molars were collected and DPCs were isolated from healthy young adults (13–28 years) undergoing orthodontic treatment in the Department of Oral and Maxillofacial Surgery, the Affiliated Stomatology Hospital of Sun Yat-sen University, with informed consent obtained from each patient as previously described [16]. The protocols were approved by the University Ethic Committee. Briefly, DPCs were cultured in D-Modified Eagle's Medium (DMEM, GIBCO-BRL Life Technologies, Breda, Netherlands) supplemented with 20% fetal bovine serum (FBS, Invitrogen, CA, USA), 10 U/mL penicillin, and 10 mg/mL streptomycin (Sigma, St. Louis, MO, USA) and incubated at 37°C in 5% CO_2_. The medium was changed every 3 days.

### 2.2. Synthesis of PEG-PEI Nanoparticles

Polyethylene glycol-polyethyleneimine (PEG 2 kDa, PEI 25 kDa) was synthesized according to the procedures as previously reported [17]. Briefly, PEG-PEI was synthesized through adding 1.25 g of hyperbranched polyethyleneimine and 0.5 g of mPEG-N-hydroxysuccinimide (NHS, 2 mmol) to phosphate-buffered saline (pH 7.4) and magnetically stirred at room temperature overnight. The solution was purified by membrane dialysis (3.5 kDa) in distilled water for 48 h and lyophilized to obtain the PEG8k-PEI25k product. PEG-PEI was characterized by 1H-NMR (Varian Mercury 30 mHz NMR spectrometer, Mountain View, CA, USA) in deuterium oxide. Fourier transform infrared measurement was carried out by Fourier transform infrared analyzer (Nicolet/Nexus 670, Woodland, CA, USA) by the KBr method.

### 2.3. Preparation of miR-146a Loaded PEG-PEI Nanoparticles

miR-146a/PEG-PEI nanoparticles were prepared and the physical characteristics were analyzed. Briefly, a predetermined (3 *μ*L 20 *μ*M) amount of miRNA was added to a vial containing the cationic micelle solution. Various micelle concentrations were used in the preparation, according to the designed nitrogen-to-phosphorus (N/P) ratio. The mixture was vortexed and maintained at room temperature for 30 m and then stored at 4°C before use.

### 2.4. Determination of the Particle Size and Zeta Potential of miR-146a/PEG-PEI Nanoparticles with Various N/P Ratio

The particle size and zeta potential of the prepared miR-146a/PEG-PEI nanocomplexes were determined by dynamic light scattering (DLS). Measurements were performed at 25°C on BI-200 SM equipment (Brookhaven Instruments Corporation, NY, USA). For the zeta potential measurement, a standard electrophoresis minicell from Brookhaven was used. The data for particle size and zeta potential were collected on an autocorrelator with a detection angle of scattered light at 90°. All measurements were conducted in triplicate and averaged to yield the mean particle size and zeta potential. Prior to zeta potential analysis standard control samples were run on the instrument.

### 2.5. Agarose Gel Retardation Assay

In order to evaluate the miRNA complexation ability of the delivery agents, gel electrophoresis was performed. The 3 *μ*L 20 *μ*M of miRNA was mixed with PEG-PEI complexes prepared at designated N/P ratios (0, 0.1, 0.2, 0.5, 1.0, 2.0, and 5.0) without dilution. Loading buffer was added to the samples. The samples were loaded onto 1% agarose gel stained with 2.0 *μ*L ethidium bromide solution and electrophoresed at 100 V for 30 m. An ultraviolet image station (Olympus, Tokyo, Japan) was used to record the gel images.

### 2.6. Evaluation of Transfection Efficiency by Flow Cytometry

Flow cytometry analysis was employed to quantify the transfection efficiency of nanoparticles in DPCs. DPCs at passage 3 were cultured in 6-well plates at a density of 2 × 10^5^/well for 24 h before transfection until they reached 60%–70% confluence. The cy3-miRNA/PEG-PEI nanoparticles and cy3-miRNA negative control were synthesized by RiboBio Co., Ltd. (RiboBio, Guangzhou, China). Briefly, the process of oligonucleotide synthesis is implemented as automated solid-phase synthesis using phosphoramidite method as previously reported [18]. miR-146a was diluted in acetate buffer, and chitosan solution was added and stirred for 1 h at room temperature. Particles were formulated by cy3-labeled miRNA specific to enhanced green fluorescent protein (eGFP). The experiment groups were incubated in cy3-miRNA/PEG-PEI nanoparticles with the N/P ratio of 0.5, 2, 5, and 20 in 400 *μ*L PBS (pH 7.4), respectively. Lipofectamine 2000 (lipo2000, Invitrogen, Carlsbad, CA, USA) was used as positive control miRNA delivery agent, and normal medium served as negative control. The amount of each sample was 100 pmol. After 6 h incubation, DPCs were harvested by trypsinization, washed in cold PBS, and fixed in 70% alcohol for 30 min on ice. After washing in cold PBS three times, cells were analyzed on FACSCalibur flow cytometer (BD Biosciences, San Jose, CA, USA), using 548 nm laser for excitation. 10^6^ cells were analyzed in each group. Normally cultured cells without nanocomplex transfection were used as negative control for background calibration. The percentage of cells internalized with nanocomplex or lipo2000 was determined by the number of cy3-positive cells, and the transfection efficiency (%) was analyzed using FCSExpress software.

### 2.7. Cellular Uptake of Nanoparticles Observed by Fluorescence Microscope

To determine the cellular uptake of nanoparticles, DPCs were cocultured with cy3-labeled miRNA/PEG-PEI nanoparticles, and the absorption ratio was observed under fluorescence microscope. DPCs were seeded in 6-well plates at a density of 2 × 10^5^/well. The nucleuses of cells were stained with Hoechst 33342 for 10 m and washed in PBS for 3 times. cy3-miRNA/PEG-PEI was prepared at designated N/P ratio of 2. DPCs were incubated with the solution for 0, 1, 2, and 4 h. The images were captured under microscope (Axiovert, Zeiss, Germany) to observe the cellular uptake of nanoparticles.

### 2.8. Cell Viability Assay

DPCs were cocultured with miR-146a/PEG-PEI nanoparticles for 48 h; their cytotoxicities on DPCs were analyzed by CCK8 quantitative assay (Cell Counting Kit-8, Dojindo, Kumamoto, Japan). DPCs were cultured in 96-well plates at an initial density of 3 × 10^3^/well with normal DMEM medium for 24 h prior to PEG-PEI induction. The experiment groups were incubated in cy3-miRNA/PEG-PEI nanoparticles with the N/P ratio of 0.5, 1, 2, 5, 10, 20, 40, and 80, respectively. Normal medium served as control. After 48 h incubation, cell viability was evaluated by CCK8 according to manufacturer's instructions. Briefly, 10 *μ*L of CCK8 solution was added to the culture medium and incubated for additional 2 h. The absorbance was determined at 450 nm wave length and the cell viability was calculated according to the formula: cell viability = (experiment group OD value/control group OD value) × 100%.

### 2.9. Synthesis and Combination of Alginate Gel with miR-146a/PEG-PEI and bFGF

The alginate was synthesized as previously described [13]. Briefly, alginate solution was prepared by dissolving 10 mg of sodium alginate (FMC Biopolymer, Philadelphia, PA, USA) in 1 mL of distilled water. After 30 min of ultrasonic stirring, miR-146a/PEG-PEI (50 *μ*L miR-146a/PEG-PEI nanoparticles), bFGF (20 *μ*L bFGF), and miR-146a/bFGF/PEG-PEI (50 *μ*L miR-146a/PEG-PEI nanoparticles and 20 *μ*L bFGF) were added to the solution, respectively. 0.666 g CaCl_2_ was added to distilled water and made into 20 mL 0.3 M solution with ultrasonic stirring. The alginate was mixed with the solution and incubated for another 2 h. The alginate beads were purified in distilled water for 3 times, and the extra solution was abandoned.

### 2.10. The Drug Release Rate by Ultraviolet Spectrophotography

The release profile of cy3-conjugated miR-146a/bFGF from the hydrogel was measured using ultraviolet spectroscopy. The hydrogel samples were incubated in PBS with pH = 7.4 at 37°C for up to 24 h. Three-millilitre supernatants at the desired time points (0, 1, 2, 3, 4, 5, 6, 8, 10, 12, and 24 h) were collected and analyzed. Relative OD values were converted into the actual weight of protein released by correlation with a standard curve.

### 2.11. Cell Proliferation Assay

DPCs were cocultured with prepared hydrogel in each group for 2 d and then incubated in 1 *μ*g/mL LPS for 4 h. DPCs of control group (DPCs cultured in normal medium), LPS group (DPCs with LPS stimulation), miR-146a gel group (DPCs cocultured with miR-146a/PEG-PEI alginate hydrogel and LPS stimulation), bFGF gel group (DPCs cocultured with bFGF alginate hydrogel and LPS stimulation), and miR-146a/bFGF gel group (DPCs cocultured with miR-146a/bFGF/PEG-PEI alginate hydrogel and LPS stimulation) were cultured in 96-well plates with DMEM supplemented with 10% FCS and 50 mg/mL gentamycin. Culture medium was changed every 3 d. Cell proliferation was determined by CCK8 quantitative assay (Cell Counting Kit-8, Dojindo, Kumamoto, Japan) according to the absorbance (450 nm) of reduced 2-(2-methoxy-4-nitrophenyl)-3-(4-nitrophenyl)-5-(2,4-disulfophenyl)-2H-tetrazolium (WST-8).

### 2.12. Effect of miR-146a/bFGF/PEG-PEI Alginate Hydrogel on Odontogenic Differentiation of DPCs with Inflammation

DPCs of each group were induced in odontogenic differentiation medium containing DMEM supplemented with 15% FBS, 10 mmol/L *β*-glycerophosphate, 0.2 mmol/L ascorbate-2-phosphate, and 100 nmol/L dexamethasone (Sigma, St. Louis, MO, USA) for 7 and 14 days. The mineral deposits were detected by Alizarin red staining. Western blot was used to determine the protein expression of odontogenic differentiation markers DMP-1 and DSP.

For Alizarin red staining, cells were fixed with 4% paraformaldehyde for 15 min at room temperature and washed with distilled water. 1% Alizarin red solution was added to the fixed cells and incubated for 20 min at room temperature and then washed with distilled water. The stain was desorbed with 10% cetylpyridinium chloride (Sigma, St. Louis, MO, USA) for 1 h. The solution was collected and distributed at 200 mL/well on a 96-well plate, and absorbance readings were taken at 590 nm using a spectrophotometer. Alizarin red levels were normalized to the total protein content.

Western blot was performed as described previously [16]. Briefly, the total protein was measured by a Bio-Rad Coomassie Blue protein assay (Bio-Rad Laboratories, Richmond, CA, USA). Twenty micrograms of protein was diluted by 10% bromophenol blue and boiled before being separated by sodium dodecyl sulfate-polyacrylamide gel electrophoresis (SDS-PAGE) and transferred to a nitrocellulose membrane. The membranes were blocked in 5% low-fat milk at room temperature for 1 h, rinsed, and incubated with monoclonal antibodies mouse against human DMP-1 (1 : 500 dilution, Santa Cruz, CA, USA), rabbit against human DSP (1 : 500 dilution, Santa Cruz, CA, USA), or human *β*-actin (1 : 1000 dilution, Santa Cruz, CA, USA) overnight at 4°C. After washing, the membrane was incubated with the HRP-conjugated secondary antibody (1 : 5000 dilution, Jackson ImmunoResearch, PA, USA) at room temperature for 1 h. Immunoreactive proteins were then visualized by incubating membranes with electrogenerated chemiluminescence plus detection agents (GE Healthcare, NJ, USA).

### 2.13. Statistical Analysis

All experiments were repeated at least in triplicate. The SPSS19.0 software package (SPSS Inc., Chicago, IL) was used for the statistical tests. Student's *t*-test was applied to compare two samples. One-way analysis of variance (ANOVA) was applied to compare the differences among multiple samples. If equality of variances could be assumed, Bonferroni test was performed. If equality of variances could not be assumed, which was also concluded by a hypothesis test, Kruskal-Wallis test was performed. The difference was considered as being of statistical significance at *p* < 0.05.

## 3. Results

### 3.1. Synthesis of PEG-PEI Nanoparticles

**Figure 1:**
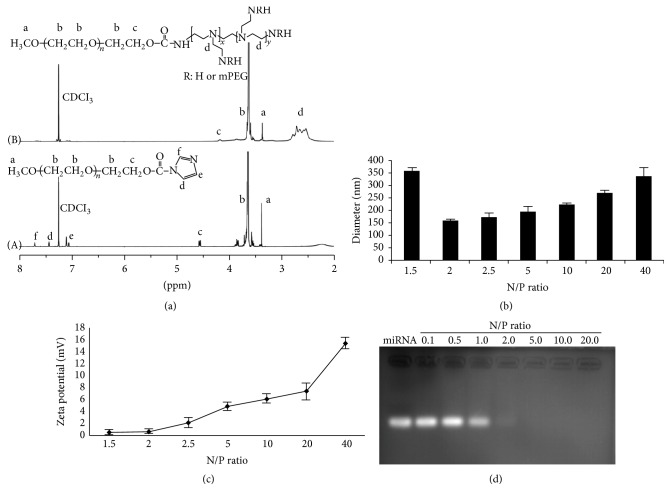
Synthesis and characteristics of PEG-PEI. (a) Nuclear magnetic resonance graph of the products: (A) mPEG-CDI, (B) mPEG-PEI. Strong characteristic peaks of PEG and PEI were shown to reside at about 3.65 ppm (peak b) and 7.35 ppm (peaks d and e), respectively. (b) Dynamic diameter distribution showed that miR-146a/PEG-PEI nanoparticles were in the range 150–360 nm at N/P ratio 1.5 to 40. The particle diameter of the nanoparticles was the lowest at the N/P ratio of 2 and maintained consistent upregulation along with the increasing of N/P value. (c) The zeta potentials were measured to 0 mV at the N/P ratio of 2 and upregulated with the increasing of N/P value. (d) Agarose gel electrophoresis image revealed that the electrophoretic mobility was partially neutralized and the bands were weakened with the increase of N/P value. The motion was completely retarded at N/P ratio of 2.

PEG-PEI was synthesized through the conjugation of PEG-NHS to PEI.  Figure 1(a) showed that strong characteristic peaks of PEG and PEI reside at about 3.65 ppm (peak b) and 7.35 ppm (peaks d and e), respectively, indicating that PEG was conjugated to the PEI chain.

### 3.2. Dynamic Diameter Distribution, Zeta Potential, and Electrophoretic Mobility of miRNA-146a/PEG-PEI

Dynamic diameter distribution, zeta potential, and electrophoretic mobility of miRNA-146a/PEG-PEI were evaluated (Figures 1(b)–1(d)). Dynamic diameter distribution showed that miRNA-146a/PEG-PEI nanoparticles were in the range 150–360 nm at N/P ratio 1.5 to 40. The particle diameter of the nanoparticles reached the lowest of 160 ± 5.37 nm at the N/P ratio of 2 and maintained consistent upregulation with the increasing of N/P value (Figure 1(b)). Similarly, the zeta potentials were measured to about 0 mV at the N/P ratio of 2 and upregulated with the increasing of N/P value (Figure 1(c)). Agarose gel electrophoresis image revealed that the electrophoretic mobility was partially neutralized and the bands were weakened with increase of N/P value. The motion was completely retarded at N/P ratio of 2, indicating full neutralization of the miRNA negative charge (Figure 1(d)). PEG-PEI nanoparticles with low toxicity and satisfied biocompatibility are preferable for gene delivery. The miRNA-146a/PEG-PEI nanoparticles prepared at N/P 2 showed complete neutralization, smallest particle sizes and zeta potential; thus N/P 2 was chosen for the following biological experiments.

### 3.3. Transfection Efficiency of miRNA-146a/PEG-PEI and miR-146a/lipo2000

**Figure 2:**
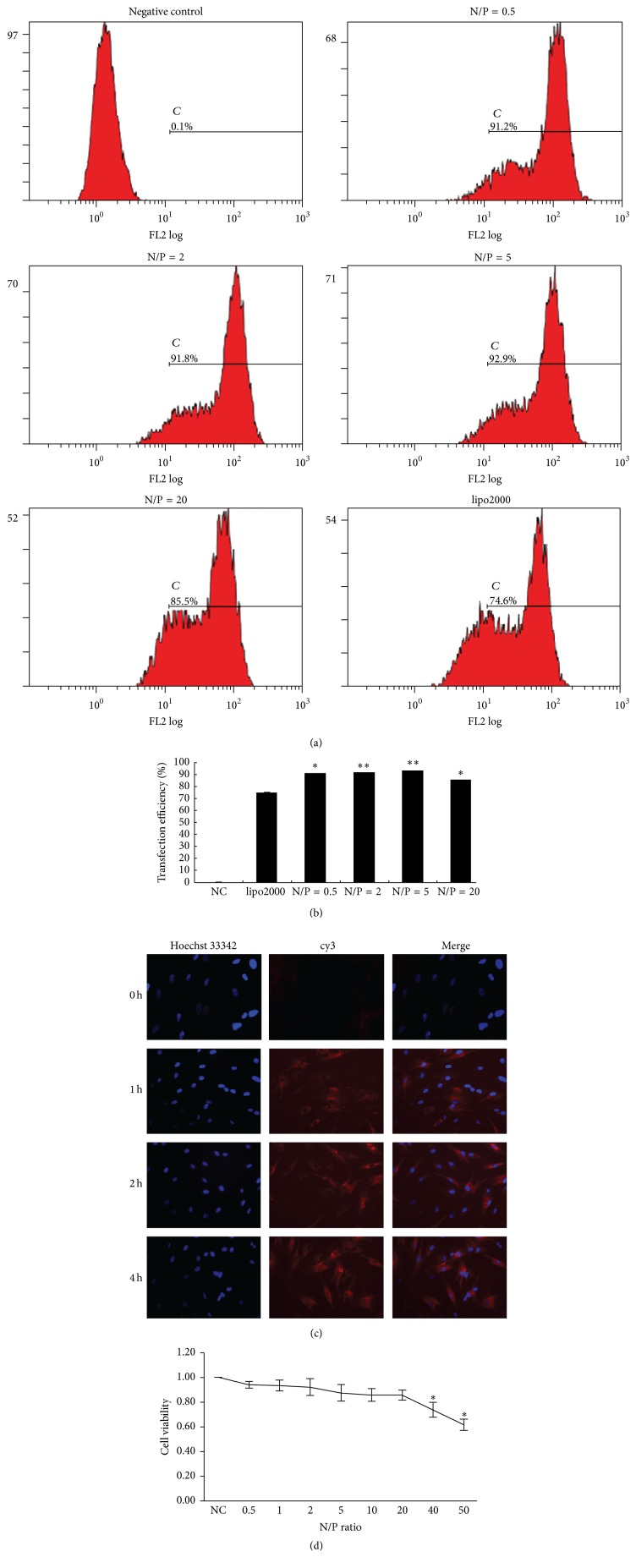
Transfection efficiency, cell uptake, and viability of DPCs incubated with miR-146a/PEG-PEI nanoparticles. ((a) and (b)) Flow cytometry showed that transfection efficiency increased along with the upregulation of N/P and reached the peak (92.9% ± 2.4%) at N/P ratio of 5 and then downregulated at N/P 20. Transfection efficiency percentages of miR-146a/PEG-PEI nanoparticles were all over 80%, which were higher than that of lipo2000 (^*∗*^ *p* < 0.05, ^*∗∗*^ *p* < 0.001). (c) Cell uptake of DPCs incubated with cy3-miRNA/PEG-PEI nanoparticles. Fluorescent images showed that no cy3-label red fluorescence was expressed in cy3-miRNA/PEG-PEI solution after 0 h incubation. After 1 h of incubation, cytoplasm of a few DPCs revealed red fluorescence, which showed the intracellular distribution of miRNA and delivery agents, whereas, after 2 h and 4 h, the number of cy3-positive cells increased and the DPCs showed stronger red fluorescence in comparison with the 0 h and 1 h groups (×200). The nucleus of DPCs was stained with Hoechst 33342 (blue fluorescence). (d) Cell viability of DPCs treated with miR-146a/PEG-PEI nanoparticles downregulated gradually along with the increasing of N/P ratio. There were no significant differences between the experiment groups and control group when the N/P ratio was lower than 20 (*p* > 0.05). However, at N/P ratio of 40 and 80, the cell viability decreased to 71.23 ± 1.67% and 63.45 ± 2.03%, respectively, which were significantly downregulated comparing with the control group (^*∗*^ *p* < 0.05).

Transfection efficiency of miRNA-146a/PEG-PEI nanoparticles in DPCs was evaluated with flow cytometry. Lipofectamine 2000 was used as positive control. The transfection efficiencies appeared different at various N/P ratios. As shown in Figures 2(a) and 2(b), transfection efficiency increased along with the upregulation of N/P and reached the peak (92.9% ± 2.4%) at N/P ratio of 5 and then downregulated at N/P 20. Transfection efficiency percentages of miRNA-146a/PEG-PEI nanoparticles were all over 80%, which were higher than that of lipo2000 (76%) and negative control group (0.9%) (*p* < 0.05), while there is no statistical difference between these four experiment groups (*p* > 0.05). These results indicated that the PEG-PEI transferred more miRNA into cells than lipo2000; thus PEG-PEI might be a valid gene delivery agent with better transfection efficiency in DPCs than lipo2000.

### 3.4. Cell Uptake of cy3-miRNA-146a/PEG-PEI Nanoparticles

The cell uptake of the cy3-miRNA-146a/PEG-PEI nanoparticles was investigated at 0, 1, 2, and 4 h after transfection using fluorescent microscopy. To evaluate the internalization of miRNA into cells, cy3-labeled scrambled miRNA was used (red fluorescence). The nucleus of DPCs was stained with Hoechst 33342 (blue fluorescence). No red fluorescence was expressed in negative control group (data not shown) and cy3-miRNA-146a/PEG-PEI solution group after 0 h incubation. After 1 h of incubation, cytoplasm of a few DPCs revealed red fluorescence, which showed the intracellular distribution of miRNA and delivery agents. After 2 h and 4 h, the number of cy3-positive cells increased and the DPCs showed stronger red fluorescence in comparison with the 0 h and 1 h groups (Figure 2(c)), indicating that more miRNA was successfully shuttled into cells by PEG-PEI nanoparticles.

### 3.5. Cell Viability Analysis

The influence of miR-146a/PEG-PEI on cell viability was examined by CCK8. DPCs were exposed to miR-146a/PEG-PEI for 48 h at N/P ratios of 0.5, 1, 2, 5, 10, 20, 40, and 80 as shown in Figure 2(d). Cells incubated in normal medium without treatment were served as control, and the cell viability value was set at 100%. There was no significant difference of cell viability between the experiment groups and control group when the N/P ratio is lower than 20 (*p* > 0.05). However, at N/P ratio of 40 and 80, the cell viability decreased to 71.23 ± 1.67% and 63.45 ± 2.03%, respectively, which were significantly downregulated compared with the control group (*p* < 0.05). This result demonstrated that the cytotoxicity increased along with the upregulation of N/P value.

### 3.6. Drug Release Profile of Alginate Hydrogel System

**Figure 3:**
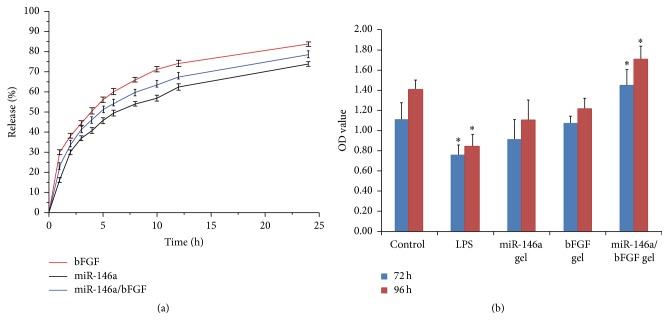
Drug release profile and cell proliferation of DPCs with LPS treatment. (a) The* in vitro* drug release experiment showed that the drug release of bFGF, miR-146a, and miR-146a/bFGF amounted to more than 70% at the beginning of the 12 h and maintained slow release over 25 h. (b) CCK8 showed that, comparing with the control group, the OD value of LPS group significantly decreased at both 72 and 96 h after treatment (^*∗*^ *p* < 0.05). Both miR-146a gel and bFGF gel groups did not show any significant difference (*p* > 0.05), albeit the OD value of miR-146a/bFGF gel group significantly increased at both 72 and 96 h after treatment (^*∗*^ *p* < 0.05).

After combining alginate gel with miR-146a/PEG-PEI nanoparticles and bFGF, the drug release profile was examined. The* in vitro* release experiment showed that the drug release of bFGF, miR-146a, and miR-146a/bFGF amounted to more than 70% at the beginning of the 12 h and maintained slow release over 25 h (Figure 3(a)). This result indicated that alginate hydrogel system is able to induce a sustained release of bFGF, miR-146a, and miR-146a/bFGF.

### 3.7. Effect of miR-146a/bFGF/PEG-PEI Alginate Hydrogel System on Cell Proliferation of DPCs with Inflammation

As shown in Figure 3(b), CCK8 results revealed that, comparing with the control group, the OD value of LPS group significantly decreased at both 72 and 96 h after treatment (*p* < 0.05), indicating that LPS could restrain the cell proliferation of DPCs. Both miR-146a gel and bFGF gel groups did not show any significant difference (*p* > 0.05). However, the OD value of miR-146a/bFGF gel group significantly increased at both 72 and 96 h after treatment (*p* < 0.05), suggesting that miR-146a/bFGF/PEG-PEI alginate hydrogel could effectively promote cell proliferation of DPCs with LPS treatment.

### 3.8. Effect of miR-146a/bFGF/PEG-PEI Alginate Hydrogel System on Odontogenic Differentiation of DPCs with Inflammation

**Figure 4:**
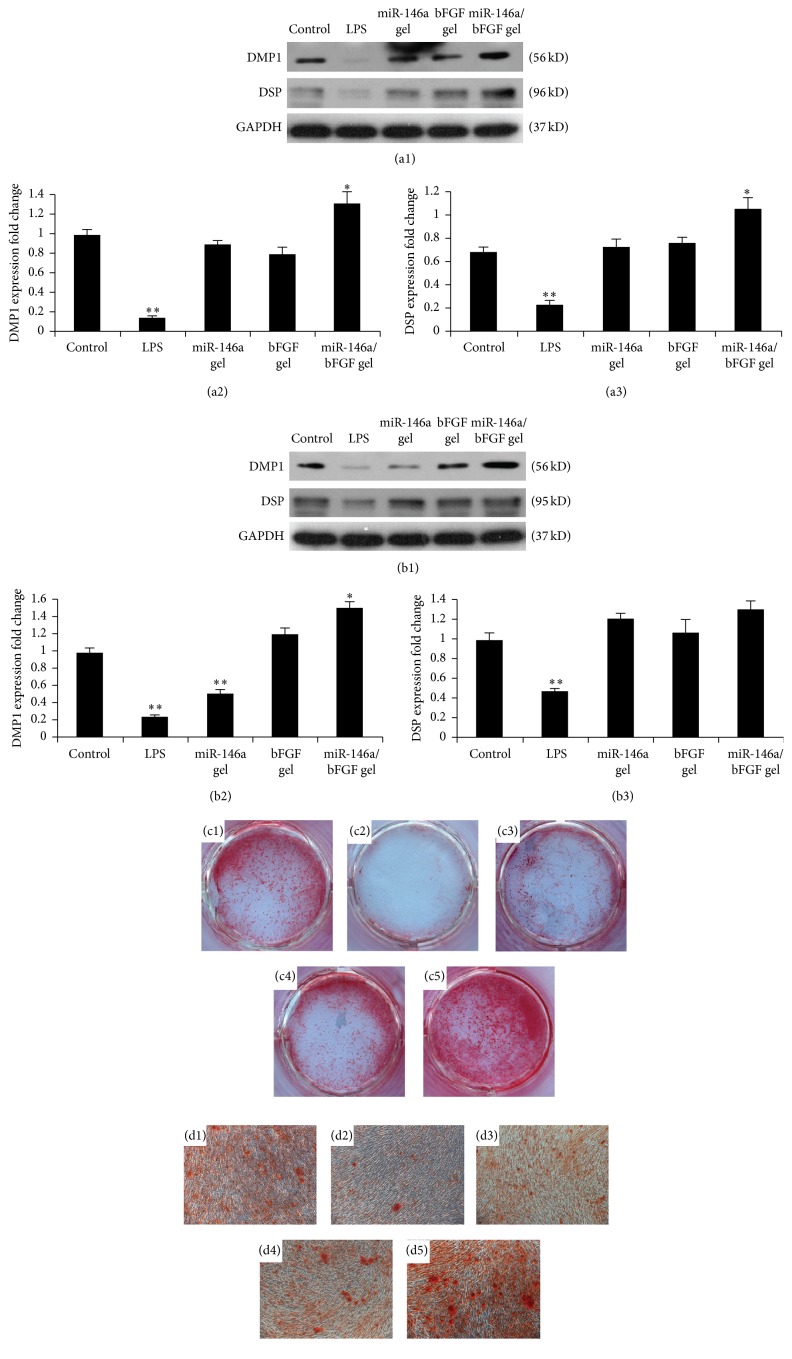
Effect of miR-146a/bFGF/PEG-PEI alginate hydrogel on the odontogenic differentiation of DPCs with LPS treatment. ((a1)–(b3)) Western blot showed that the protein expression of DMP-1 and DSP was significantly downregulated in DPCs with LPS treatment after 7 d and 14 d LPS treatment compared with control group (^*∗∗*^ *p* < 0.001). After 7 d of odontogenic induction, both DMP-1 and DSP did not show any significant difference in miR-146a gel and bFGF gel group ((a1)–(a3), *p* > 0.05), albeit dramatically upregulated in miR-146a/bFGF gel group ((a1)–(a3), ^*∗*^ *p* < 0.05). After 14 d of odontogenic induction, DMP-1 expression was significantly downregulated in miR-146a gel group (^*∗∗*^ *p* < 0.001) and upregulated in miR-146a/bFGF gel group (^*∗*^ *p* < 0.05), albeit showing no significant difference in bFGF gel group ((b1)-(b2), *p* > 0.05), whereas DSP did not show any significant difference in miR-146a gel and bFGF gel group and miR-146a/bFGF gel group ((b1) and (b3), *p* > 0.05). ((c1)–(d5)) Alizarin red staining revealed that, after 14 d of odontogenic induction, numerous mineralization deposits were seen in all groups. The number of mineral nodules was downregulated in LPS group ((c2) and (d2)) compared with control group ((c1) and (d1)). The number of mineral nodules showed no difference between the miR-146a gel ((c3) and (d3)), bFGF gel ((c4) and (d4)), and control group ((c1) and (d1)), albeit the number and average size of mineral nodules were upregulated in miR-146a/bFGF gel group ((c5) and (d5)). ((c1)–(c5)) The gross view of the samples with Alizarin red staining. ((d1)–(d5)) The figures of the samples with Alizarin red staining observed under inverted microscope (×100). ((c1) and (d1)) Control group, ((c2) and (d2)) LPS treated group, ((c3) and (d3)) miR-146a gel group, ((c4) and (d4)) bFGF gel group, and ((c5) and (d5)) miR-146a/bFGF gel group.

Western blot showed that the protein expression of DMP-1 and DSP revealed a similar expression pattern, which was significantly downregulated in DPCs with LPS treatment after 7 d and 14 d of LPS treatment compared with control group (Figures 4(a1)–4(b3), *p* < 0.001). After 7 d of odontogenic induction, both DMP-1 and DSP did not show any significant difference in miR-146a gel and bFGF gel group (Figures 4(a1)–4(a3), *p* > 0.05), while DMP-1 and DSP were dramatically upregulated in miR-146a/bFGF gel group (Figures 4(a1)–4(a3), *p* < 0.05). After 14 d of odontogenic induction, DMP-1 expression was significantly downregulated in miR-146a gel group (*p* < 0.001) and upregulated in miR-146a/bFGF gel group (*p* < 0.05), albeit showing no significant difference in bFGF gel group (Figures 4(b1) and 4(b2), *p* > 0.05), whereas DSP did not show any significant difference in miR-146a gel, bFGF gel, and miR-146a/bFGF gel groups (Figures 4(b1) and 4(b3), *p* > 0.05).

The Alizarin red staining revealed that, after 14 d of odontogenic induction, numerous mineralization deposits were seen in all groups (Figures 4(c1)–4(d5)). The number of mineral nodules was downregulated in LPS group (Figures 4(c2) and 4(d2)) compared with control group (Figures 4(c1) and 4(d1)). However, the number of mineral nodules showed no difference among the miR-146a gel (Figures 4(c3) and 4(d3)), bFGF gel (Figures 4(c4) and 4(d4)), and control group (Figures 4(c1) and 4(d1)). The number and average size of mineral nodules were upregulated in miR-146a/bFGF gel group (Figures 4(c5) and 4(d5)). These results indicate that miR-146a/bFGF/PEG-PEI alginate hydrogel is effective for promoting the odontogenic differentiation capability of DPCs with inflammation.

## 4. Discussion

MicroRNAs (miRNAs) are small noncoding nucleotides negatively regulating protein-coding gene expression at the posttranscriptional level. Various studies have demonstrated that miRNAs play important roles in the regulation of inflammatory and immune response triggered by bacterial sensing [19]. The miR-146 family consisted of two evolutionary conserved miRNA genes, miR-146a and miR-146b, locating on chromosomes 5q33.3 and 10q24.32, respectively, in human. miR-146a has been shown to be involved in cell proliferation, differentiation, apoptosis, inflammatory response, and extracellular matrix metabolism [4, 5, 19]. Previous study reported a 5- to 25-fold increase in the expression of miR-146a and migration in DPCs with LPS stimulation, and LPS is able to increase the migration of DPCs by modulating the miR-146a-TRAF6/IRAK1 regulatory cascade [20]. miR-146a is involved in regulation of the immune response stimulated by LPS through negatively regulating NF-*κ*B, which targets IRAK-1 and TRAF-6, resulting in reduction of proinflammatory cytokines TNF-a, IL-6, IL-1b, and MCP-1 [5]. miR-146a was upregulated in inflamed gingival tissue, downregulated the TLR signaling, and negatively regulated the innate immune response [21]. However, since expression and immune regulation of miRNAs exhibit tissue-specific pattern [19], the underlying mechanism of miRNAs regulating inflammation process and regeneration capability of DPCs remains unknown.

Liposome and polycationic polymer-based nonviral systems are common nonviral carrier with high biocompatibility and little immunogenicity for delivering miRNA or siRNA* in vitro* [10]. Compared with liposome, polymer-based nonviral vectors have advantages in safety, potential large-scale production, and physical stability [12, 22]. It is reported that PEI produced transfection efficiency of 19% at N/P ratio of 8 in human adipose derived stem cells, which was higher than that achieved by Lipofectamine [23]. PEG, with increased biocompatibility, is also frequently applied to the coating of magnetic nanoparticles [11]. In the present study, liposome, as a commonly used commercial nonviral gene delivery vehicle, was used as a positive control for the evaluation of transfection efficacy. Our results confirmed that the transfection efficiency of PEG-PEI nanoparticles at various N/P values was significantly higher compared to lipo2000, which agreed with the previous report [23].

Small size and low positive charge are beneficial for easy cell uptake of nanoparticles, since low positive charge could maintain the stability of nanoparticles* in vivo*, while high positive charge nanoparticle may interact easily with negatively charged molecules [24]. In the present study, cell viability was above 85% at N/P of 0 to 20, demonstrating the low cytotoxicity and high biocompatibility of miR-146a/PEG-PEI nanoparticles. Moreover, miR-146a/PEG-PEI nanoparticles prepared at N/P 2 showed the complete neutralization, smallest particle sizes, and zeta potential; thus N/P 2 was chosen for the following biological experiments.

bFGF is involved in development, inflammation, wound healing, cell proliferation, migration, osteogenic differentiation, angiogenesis, and extracellular matrix formation [6–8]. It is known that exogenous bFGF could maintain self-renewal, high proliferation, and multilineage differentiation potential of stem cells [25]. bFGF enhances stemness property by increasing stem cell gene expression, cell proliferation, and multilineage differentiation potency of stem cells from the apical papilla (SCAP) [26]. bFGF induced* in vivo* recellularization and revascularization in endodontically treated human teeth, and DPCs could be recruited and subsequently differentiated by stimulation of bFGF in the regeneration of dental pulp [27]. Moreover, bFGF, serving as a wound healing factor in pain sensitization, was released during inflammation and tissue regeneration [28]. The concentration of bFGF was increased in arthritis rat models and patients, as well as inflammatory bowel disease [29]. Expressions of inflammation related molecules, including MMP-1, MCP-1, IL-1ra, bFGF, and VEGF, were enhanced by costimulation with proinflammatory cytokines IL-1*β* and IL-6 in human gingival fibroblasts during gingival inflammation [30]. However, the short half-life value and easy degradation make it difficult to maintain the slow release and sustain the effect of bFGF on wound healing; thus it is necessary to apply a gene delivery system.

Alginate, a linear heteropolysaccharide with high biocompatibility, is often used to generate hydrogel for the delivery of drugs or proteins, and so forth. Alginate hydrogel provides a suitable environment for tissue regeneration and cell migration [13, 14]. It is reported that heparin and alginate gel maintained long term release of bFGF under physiological conditions* in vitro* [31]. Combined with the advantages of PEG-PEI nanoparticles, a new PEG-PEI alginate hydrogel complex was successfully developed. For the first time, the alginate gel was used as a carrier to combine miR-146a/PEG-PEI nanoparticles and bFGF and demonstrated that miR-146a/bFGF/PEG-PEI alginate hydrogel is effective for promoting the cell proliferation and odontogenic differentiation of DPCs with inflammation. This result implies that a well-designed PEG-PEI alginate hydrogel nanocomplex could provide a microenvironment beneficial for improving tissue regeneration capability of DPCs in response to inflammation. Therefore, PEG-PEI alginate hydrogel could serve as a promising delivery vector for gene delivery in dental regeneration.

Furthermore, miR-146a/bFGF gel significantly enhanced cell proliferation at various time points in DPCs with LPS treatment and promoted odontogenic differentiation capability through upregulating expression of DMP-1, DSP and the formation of mineral nodules. However, miR-146a gel and bFGF gel group did not show significant effect on proliferation and differentiation of DPCs. It might be possible that miR-146a decreased the secretion of inflammation cytokines, subsequently providing a microenvironment beneficial for the positive effect of bFGF on odontogenic differentiation and tissue regeneration. Therefore, miR-146a and bFGF might work cooperatively to promote the regeneration capability of DPCs in response to inflammation, whereas miR-146a or bFGF solely is not sufficient to initiate this biological process.

Taken together, the present study revealed that PEG-PEI, with low cytotoxicity, cellular adsorption properties, constant protein release ratio, and high transfection efficiency, is a promising nonviral carrier for potential gene therapy. miR-146a/bFGF play an essential role in enhanced cell proliferation and odontogenic differentiation of DPCs in response to inflammation, indicating that miR-146a and bFGF might play a critical cooperative role in the modulation of inflammation response and regeneration of DPCs. New insights into the interplay of inflammation response and tissue regeneration will open novel therapeutic opportunities for dental patients.
